# Factors impacting clinical data and documentation quality in Australian aged care and disability services: a user-centred perspective

**DOI:** 10.1186/s12877-024-04899-1

**Published:** 2024-04-12

**Authors:** Gap Tshering, Lakkhina Troeung, Rebecca Walton, Angelita Martini

**Affiliations:** 1 Brightwater Research Centre, Brightwater Care Group, Inglewood, Australia; 2 The University of Western Australia, Crawley, Australia

**Keywords:** Health information system, Data quality, Aged care, Disability, Root cause analysis, Thematic analysis

## Abstract

**Background:**

Research has highlighted a need to improve the quality of clinical documentation and data within aged care and disability services in Australia to support improved regulatory reporting and ensure quality and safety of services. However, the specific causes of data quality issues within aged care and disability services and solutions for optimisation are not well understood.

**Objectives:**

This study explored aged care and disability workforce (referred to as ‘data-users’) experiences and perceived root causes of clinical data quality issues at a large aged care and disability services provider in Western Australia, to inform optimisation solutions.

**Methods:**

A purposive sample of *n* = 135 aged care and disability staff (including community-based and residential-based) in clinical, care, administrative and/or management roles participated in semi-structured interviews and web-based surveys. Data were analysed using an inductive thematic analysis method, where themes and subthemes were derived.

**Results:**

Eight overarching causes of data and documentation quality issues were identified: (1) staff-related challenges, (2) education and training, (3) external barriers, (4) operational guidelines and procedures, (5) organisational practices and culture, (6) technological infrastructure, (7) systems design limitations, and (8) systems configuration-related challenges.

**Conclusion:**

The quality of clinical data and documentation within aged care and disability services is influenced by a complex interplay of internal and external factors. Coordinated and collaborative effort is required between service providers and the wider sector to identify behavioural and technical optimisation solutions to support safe and high-quality care and improved regulatory reporting.

**Supplementary Information:**

The online version contains supplementary material available at 10.1186/s12877-024-04899-1.

## Introduction

In Australia, aged care and disability service providers are required to maintain comprehensive administrative and clinical records for clients [1, 2]. Aged care services include both residential (facility-based) and home-based care for non-Indigenous Australians aged over 65 years and Indigenous Australians aged over 50 years [3]. Disability services include residential and home-based care for all Australians under 65 years with long-term intellectual, mental, cognitive, physical and sensory impairments [4]. In 2022, an estimated 178,000 Australians received aged care services, while an estimated 519,000 received disability services through the National Disabilities Insurance Scheme [5], constituting an essential part of Australia’s healthcare sector.

To ensure quality and safety of aged care and disability services, enhanced information reporting requirements have been introduced in recent years as part of legal, regulatory and funding obligations. Care providers are required to collect data on, but not limited to, financial and prudential matters, clinical quality indicators, care delivery, and serious incidents [6]. Reporting requirements vary according to service type within both aged care and disability based on location (residential vs. home-based care) and duration of care (long-term vs. transitional care). Service providers are therefore challenged to collect extensive clinical information to meet multiple reporting requirements.

To facilitate reporting, providers rely on routine health information collected at the point-of-care, primarily through electronic health record (EHR) systems or clinical information systems (CIS) [7]. Clinical data is defined as any information documented within the client’s EHR that supports the workforce to complete clinical and care activities for the clients, including demographic information, clinical history, clinical assessments, care notes, and medication records. These clinical data also serve as crucial inputs which enable advanced real-time analytics to support clinical decision-making and business decisions [8]. Poor data quality is a major barrier to the effective use of CIS [9] and can lead to healthcare errors [10, 11], flawed business decisions, and increased operating costs [12]. Reliable clinical data is therefore imperative for health service performance and has made data quality an essential part of information systems research and practice in recent years [13].

Optimising data quality within health services is expected to contribute to improved patient outcomes [4], support reporting requirements, and reduce operating costs. However, there is limited guidance available to direct aged care and disability service providers in what constitutes optimal documentation [14]. This is in contrast to hospital and primary care settings where standardised national guidelines for health information documentation exist [15, 16]. Further, studies aimed at understanding and improving data quality in CIS within healthcare have also primarily been conducted in hospital settings or within specialist care settings such as dementia care [13, 17–27].

Few studies addressing improving clinical data quality in aged care and disability services currently exist. Some studies have examined the impact of EHR implementation on workflow and resident health outcomes in Australian aged care facilities [28–33] and highlighted the potential to transform routinely collected aged care data for research and sector innovation [8]. These studies show that while there is widespread agreement of the benefits of CIS for improving workforce efficiency [22, 34] and resident health outcomes [35], there is a need to improve data quality to enhance care delivery [36]. However, the specific causes of data quality concerns within aged care and disability services and solutions for optimisation have yet to be explored.

While the definition of data quality can differ from the perspectives of data producers, consumers and custodians [37], *‘fitness for use’* [38] is widely used in the literature to describe data quality. This term focusses on consumer or ‘*user’* requirements of the data being captured which can encompass multiple dimensions [37]. Data-user involvement in quality improvement research is a well-established method to understand underlying problems and develop effective and sustainable quality improvement solutions from those who are directly involved in the process of interest [39, 40].

As part of the OPTIMISE study [41], this study aimed to explore aged care and disability workforce (referred to as ‘data-users’) experiences and perceived root causes of clinical data quality concerns at a large aged care and disability services provider in Western Australia (WA). The specific objectives of the study were to: (i) understand day-to-day clinical documentation workflow across different data-user groups, and (ii) identify root causes of clinical data quality issues, to inform optimisation solutions across care services.

## Methods

### Study design and setting

This study forms part of the larger OPTIMISE study [41], which is a prospective pre-post optimisation study using an integrated Agile Lean Six Sigma DMAIC (Define, Measure, Analyse, Improve, Control) framework [42] aimed at identifying opportunities for optimising the quality of clinical data across aged care and disability services operated by Brightwater Care Group (‘Brightwater’) in WA. Brightwater provides residential and home-based care for approximately *n* = 2,000 aged care and *n* = 500 disability services clients across eight different programs (Table 1) [41]. Two standalone vendor-based systems or CIS are used across care services: Clinical Manager [43] (for residential care facilities) and CareLink+ [44] (for home-based care). Details of the full study design are presented in our protocol paper [41].

**Table 1:** Overview of aged care and disability services

Service Type	Program Name	Sites, n	Clients, n	Description
Aged Care	RAC^a^	11	750	Long-term/permanent high care accommodation for people aged > 65 years
	SDCP^b^	1	31	Long-term/permanent high care accommodation for people with dementia
	TCP^c^	2	101	Short-term, post-hospital support and active management for people aged > 65 years
	AH^d^	-	1,000	Home-based support for people aged > 65 years
Disability	TRP^e^	1	53	Specialist neurorehabilitation service for people aged 18–65 years with acquired brain injury
	TAP^f^	1	23	Short-term, post-hospital support and active management for people aged 18–65 years with disability
	SIL^g^	8	71	Long-term/permanent high care accommodation for people aged 18–65 years with disability
	CAPB^h^	-	375	Home-based support for people aged 18–65 years with disability and NDIS^i^ funding

**Source**: Troeung et al., 2023

^a^RAC: residential aged care

^b^SDCP: Specialist Dementia Care Program

^c^TCP: Transitional Care Program

^d^AH: at home

^e^TRP: Transitional Rehabilitation Program

^f^TAP: Transitional Accommodation Program

^g^SIL: Supported Independent Living

^h^CAPB: capacity building

^i^NDIS: National Disability Insurance Scheme

In the *Measure* stage, a baseline audit of clinical data quality was undertaken using EHRs for *n* = 2,404 clients active in aged care and disability services between 1 July to 31 December 2021 [45]. Baseline data quality assessments used a combination of data warehouse audits and manual clinician review, to assess the quality of EHRs across six data-user-identified metrics: Completeness, Currentness, Accuracy, Clarity, Compliance, and Usability. In total, 18 key data quality issues were identified across services [45]. Baseline assessments were followed by the *Analyse* stage, which used qualitative research methodology to understand data-user experiences of Clinical Manager and CareLink+, and to explore potential root causes of clinical data quality issues in these systems.

### Participants and recruitment

Purposive sampling was used to select services for consultation. A total of 10 residential care facilities were selected for face-to-face or online interviews (5 aged care, 5 disability), while home-based care staff were consulted through an online survey. For programs with multiple facilities such as Residential Aged Care (RAC) and Supported Independent Living (SIL), we purposively selected the 3 facilities with the highest, median, and lowest data quality scores from the baseline audit [45]. Based on prior research, carrying out usability testing with 8–10 participants should identify 80% of usability problems [46].

Participant inclusion criteria were staff members who used: (1) Clinical Manager and/or CareLink + for clinical documentation (primary data-users), and/or (2) clinical data for reporting, analytics or clinical/service decision making (secondary data-users). Staff from five functional groups were included: Service managers, clinical staff, care staff, administration staff, and corporate staff.

### Data collection

#### Interviews

A semi-structured interview guide was developed, piloted and revised with clinical and research team members prior to use. Interview questions were designed to understand the underlying or ‘root causes’ of key data quality issues identified in the *Measure* stage. The final interview guide consisted of eight structured and semi-structured questions [47] (Appendix 1).

In-person visits to residential care facilities were conducted by two researchers (GT, RW) between September and October 2022. The first interviewer (GT) is an Information Systems Analyst, and the second (RW), a clinician. During each visit, convenience sampling was used to recruit eligible staff members who were available at the time for interviews. After verbal informed consent was obtained, one researcher interviewed participants, while the other recorded participant responses into a spreadsheet. Interviews were not audio recorded. Recruitment continued until all eligible staff were approached or saturation of responses was reached [48]. Online interviews were conducted over Microsoft Teams for key participants (e.g., site managers, clinical leaders) who were unavailable during the day of the visit.

#### Surveys

Additionally, an online survey, consisting of a written version of the interview questions, was created using Survey Monkey [49] and emailed to all staff providing home-based care. Surveys were used as home-based staff work directly within client homes without a central office location, thus presenting logistical challenges for conducting face-to-face interviews.

### Data analysis

Inductive thematic analysis was used to analyse interview and survey responses. This involved identifying themes from the data without depending on an established theoretical framework [50]. Inductive analysis was selected to reduce potential bias caused by one author completing the initial coding. The researchers used a combination of consensus and split coding, due to the small nature of the research team and the desire to maintain transparency and transferability in the analysis [51]. One researcher (GT) read through all participant responses to establish familiarity with the data, assign initial codes then generate subthemes and themes. Two researchers (RW, LT) independently analysed a random sample of responses (30% of all responses) to assign codes and generate themes. A hierarchical coding frame was used, creating top-level and second-level codes, which became the themes and sub-themes generated by the analysis [52]. All three researchers met following initial analysis to discuss where coding and themes did not match or where extra codes and themes were required. A consensus method was used for discrepancies. Where researchers could not agree on a theme, a fourth researcher (AM), with expertise in qualitative research and analysis, was consulted to resolve discrepancies. Once initial themes and subthemes were determined, two researchers (GT, RW) checked the findings to ensure there was adequate evidence to support the themes and subthemes. A final consensus meeting was held to ensure all researchers agreed with the developed themes and subthemes. During this meeting, quotes that strongly captured the essence of the theme were selected from a list of relevant quotes, based on consensus.

## Results

### Participant characteristics

A total of *n* = 135 staff members participated in the interviews (*n* = 84, 62%) and surveys (*n* = 51, 38%), representing approximately 6.3% of the total organisational workforce. There was a balanced composition of participants from aged care (*n* = 41, 30%), disability services (*n* = 27, 20%), community-based care (*n* = 51, 38%), and corporate staff (*n* = 16, 12%). Most participants had been employed at the organisation for less than four years (Table 2).

**Table 2:** User Consultation Participants by Functional Groups (*n* = 135)

Service	Functional User Group	Number of Participants	Average Time in Role
**Aged Care**	Service manager/Coordinator	6	< 2 years
	Clinical	13	2 to 4 years
	Allied health	5	2 to 4 years
	Therapy assistant	6	> 4 years
	Care worker	8	> 4 years
	Administration	3	> 4 years
**Disability**	Service manager/Coordinator	4	> 4 years
	Clinical	3	2 to 4 years
	Allied health	2	2 to 4 years
	Therapy assistant	1	< 2 years
	Care worker	15	> 4 years
	Administration	2	> 4 years
**Corporate**	Managers	15	2 to 4 years
	Administration	1	2 to 4 years
**BAH & CAPB** ^1^	Service manager/Coordinator	4	> 4 years
	Clinical	14	< 2 years
	Allied health	19	< 2 years
	Therapy assistant	3	< 2 years
	Care worker	2	2 to 4 years
	Administration	9	< 2 years
**Total**			

^1^ *Brightwater At Home (BAH) and Capacity Building (CAPB) represents community-based care services. Participants in this service respondent to a web-based survey*

Eight overarching themes and 29 subthemes were identified as potential causes of data quality issues in CISs (Table 3).

**Table 3:** Summary of Themes and Subthemes Representing Factors Influencing Data Quality in iCare and CareLink+

THEMES	SUBTHEMES	DESCRIPTOR	QUOTES
1. **Staff related challenges**	1.1. **Staff behaviour and practices**	Staff non-compliance and attitudes toward documentation practices, and missing details in second hand documentation (e.g. documenting on behalf of other staff)	*‘Some staff just don’t care [about documenting properly]’ (1).* *‘I request others to document on my behalf. So, I write only brief observation on a paper’ (16).*
		Verbal communication of client information between staff	*‘[A] client passing away is currently communicated verbally’ (66).* *‘I wish everything was [documented] in iCare for us, as things get forgotten at handover’ (66).*
		Unintentional human error in documentation	*‘We have a lot of documents to upload (GP or hospitals), the timeliness of info going into iCare….Transfer of information from admission documents – human error that can happen sometimes.’ (72).* *‘If it is incorrect, it means that somebody has made the wrong entry’ (77).* *‘Entering a note for the wrong client is an easy mistake to make on CareLink+’ (86).*
	1.2. **Cultural diversity**	Staff English language skills impact clear and accurate documentation	*‘There are so many free text fields where quality of notes largely depends on the level of English of staff completing those fields’ (84).*
	1.3. **Diversity in technical skills**	Staff literacy skills and computer skills impact use of clinical systems for documentation purpose	*‘It doesn’t appropriately engage the user at their level of literacy/English fluency/clinical knowledge’ (32).* *‘A lot of us were not used to computers. We learned from bits and pieces and picking other people’s brain’ (64).*
	1.4. **Diversity in clinical knowledge**	Staff level of clinical knowledge impact on data input and interpretation	*‘As I don’t have clinical background, [it is] sometimes not easy to understand clinical terms’ (57).* *‘[A lot of] Information is entered in the system which are summary text with acronyms and short-form [and] are difficult to understand. Information should be entered in a language that can be understood by other users’ (81).*
2. **Education and training**	2.1. **Role specific training**	Staff do not receive training to understand the specific requirements of their role	*‘People understanding their roles and responsibilities [impact how up to date data and documentation are]’ (25).* *‘Difficult to understand some forms and charts that are not required to be completed daily and that are not explained to us. For example, pain chart and forms’ (44).*
	2.2. **Formal training in system use**	Staff receive minimal/no training on system use and expectations	*‘There is no specific training. So, usually, the person who is new…is shown how to use iCare through someone on site’ (26).* *‘We were only educated on progress notes and charts. The rest, we were not shown. When I was on the floor, I didn’t know what they were for. It wasn’t until I was thrown into this role that I just clicked on everything’ (63).*
3. **External barriers**	3.1. **Governance of external providers**	There is no onus on external providers to enter client data into systems, relying on verbal handover	*‘What people enjoy or how they reacted to an event. NDIS providers have their own book for notes. All social activities are [provided by] external providers, so we don’t know what happens’ (41).*
	3.2. **Errors in health/medical documentation**	There are often errors in discharge information provided by hospitals and other external providers, which are transferred to clinical systems	*‘[There are] incorrect information from hospital and [we] need to redo assessment’ (5).*
	3.3. **Families as information source**	Families are contacted for background client information, but don’t always understand what is being asked of them or are unable to provide accurate information	*‘Sometimes family members don’t understand what we are trying to get out of them as admission information, which goes into better supporting clients, [so this information is missing]’ (54).*
	3.4. **Sector-wide staff shortages**	Sector-wide staff shortages create greater time constraints and more need for agency staff who aren’t familiar with systems or business procedures	*‘Short [of] staff and therefore time is an issue’ (1).* *‘There are chances some people won’t write it straight away, so it’s not always entered…. or they are in a hurry, shortage of staff, clients need attention’ (40).*
4. **Operational guidelines and procedures**	4.1. **Data and documentation requirements**	Lack of clarity of data and documentation requirement, timeline for updating client information, and standard practices when documenting client progress	*‘There are no guidelines as to what to collect or a checklist’ (34).* *‘This is not just a systems problem; Brightwater needs to know requirements clearly to make system only provides what is needed instead of [collecting] all unnecessary details. Brightwater is the culprit’ (29).* *‘[There is] Low clarity around expected format of notes - some clinicians document more in-depth than others’ (89).*
	4.2. **Staff roles and responsibilities for data collection**	Lack of clarity of staff roles for data collection (including rushed data collection on client admission)	*‘Everyone’s responsible so no one’s responsible’ (60).* *‘Expectations for demographic data and accountability for roles [are] not clearly outlined or monitored’ (32).*
	4.3. **Staff roles and responsibilities for monitoring data collection**	Lack of clarity of staff roles for monitoring data collection and checking data quality	*‘No one is monitoring update of information [to ensure data is up to date and that the information collected is of high quality]’ (63)* *‘Who would monitor it anyway, who would start it, and who does training on it’ (34).*
5. **Organizational practice and culture**	5.1. **‘One size fit all’ approach**	Business uses two clinical systems (Clinical Manager and CareLink+) to service multiple business areas with unique data and reporting requirements	*‘[iCare] may be working well for RAC but not for TCP’ (1).* *‘iCare is RAC focused, the care plans populate automatically in a way that is not appropriate for disability’ (71).* *‘There is no social worker assessment for [our site] with the right details – we have to use a word document and upload it’ (35).* *‘Actualise function is not suitable for Capacity Building’ (90).*
	5.2. **Burden of documentation**	Staff are busy and documentation is often not prioritised as a result	*‘If you have to write too many things, people copy and paste and it’s not always right (40)”.* *‘Incident form gets completed at the end of the shift as we are busy during shift hours. Often incident is forgotten at the end of the shift’ (50).* *‘I often need to back date notes due to being too busy on the day’ (35).* *‘[There are] hundreds of progress notes… [It’s] too much to look at start of the shift’ (59).*
	5.3. **Communication of system or procedure change to workforce**	Changes to systems and processes not communicated adequately	*‘[Brightwater] don’t communicate changes when they make changes to processes’ (56).* *‘Don’t put information in a big letter to inform changes. Short SMS will be more effective to inform changes, I can’t be bothered reading through the long emails from IT’ (56).*
	5.4. **Duplication of effort**	Multiple forms duplicate information, and staff are still using paper-based forms which need to be uploaded	*‘Forms doesn’t speak to each other and thus, creating duplication’ (28).* *‘Some staff still uses paper-based forms which takes time to be entered into iCare’ (82).* *‘[staff] saving [documents] to F-Drive for easy retrieval’ (37).*
6. **Technological infrastructure challenges**	6.1. **System speed, connectivity, and reliability**	Internet speed, Wi-Fi connectivity, and computer and clinical system performance impact on staff ability to complete data entry	*‘The system is slow. It plays a big part in me putting information into the system, and there is a lag during busy times’ (41).* *‘The system will time out. It will log out in the middle of a note if you move away’ (35).* *‘Last time I used CareLink + was Monday afternoon and it crashed. It’s been crashing a lot lately’ (78).*
	6.2. **Technological support**	Unclear procedure to feedback on bugs found in the system	*‘Don’t know who to contact to fix bugs [in iCare]’ (41).*
	6.3. **Equipment and Resources**	Not enough computers at sites to support uninterrupted documentation at the point-of-care	*‘Staff queue at the end of shift to document notes’ (31).* *‘IT has been the biggest issue, with the constant glitches, one day iPads aren’t working [but] desktops are working, but there are not enough computers’ (81).*
7. **Systems design limitations**	7.1. **Data extraction and reporting**	*Difficult to extract data for reporting*	*‘Functions to pull out data is difficult, and it is difficult to work with reports’ (49).* ‘*Reporting does not work. It does not pull correct data in the reporting, or it is not complete’ (52).* *‘From a backend perspective, the modelling of the data and tables at the backend makes it difficult to pull data out for reporting purposes’ (79).* *‘I think when these systems were created, they looked at it from function, rather than reporting and didn’t think about pulling data out for reporting’ (79).*
	7.2. **Search and filter**	Lack of search and filter function impact locating data and information	*‘There is no search function to look for documents by keyword’ (30).*
	7.3. **Single view of information**	Lack of single view of crucial client information	*‘[The System] should make everything available in one place rather than having to navigate through many places’ (49).*
	7.4. **Change history**	Lack of timestamps and detailed change history	*‘There is no visible history of data being up to date’ (31)* *‘I don’t think it’s possible to tell which field was updated by which person on this date’ (81).* *‘Can’t tell [if information has been updated] as there is no timestamp. Also, it’s hard to tell who edited the information as it doesn’t show the name of the person who edit data’ (49).*
8. **Systems configuration**	8.1. **Dropdown Values**	Generic dropdown values which do not capture sufficient clinical detail	*‘Not everything fits into the dropdown’ (7).* *‘[The client is] Blind in the right eye for example – iCare dropdown only has sight/vision [issue]. Dropdown [values] limits and prevents person-centred care’ (4).* *‘Yes, staff often find it difficult to understand what to choose from dropdown list. E.g., Client incident form’ (31).* *‘The dropdown is good for quickly getting things done but there is no point as all clients have generalised points but certainly not specific to person’ (6).*
	8.2. **System configuration for free text fields**	Overreliance on unstructured free-text fields	*‘There are so many free text fields where quality of notes largely depends on the level of English of staff completing those fields’ (84).* *‘Carer documentation is very freehand and so quality isn’t amazing’ (25).* *‘Information is put into free text fields, hard to be able to pull information out’ (26).* *‘It’s unclear on what to write in the free text fields’ (28).*
	8.3. **System configuration for information mapping**	Inadequate information mapping and errors in automation	*“I tell staff to pretend it’s Fifty First Date and start from scratch each time” (29).* *‘Assessments don’t create alerts automatically and that’s why the reviews [are] forgotten when [staff] are busy’ (2).* *‘Second wound entry overwrites active wound reminder’ (60).* *‘Cognition assessment that are mapped to care plan can’t be updated in care plan (for small changes). We need to go to the cognitive assessment to add small changes…. Interventions are not going to care plan.’ (9)*
	8.4. **System configuration for unused capabilities**	Underutilisation of system capabilities	*‘There are more features that we don’t know of’ (47).* *‘There are more data in iCare that we could use but we don’t have training to be able to use all of its features’ (51).* *‘It’s a waste if we are not using it properly’ (33).*
	8.5. **Integration of multiple systems and information transfer**	Internal data systems do not facilitate the effective two-way flow of information	*‘I understand there are still glitches between e10 and iCare. So, sometimes it doesn’t transfer across very well. Sometimes Admin will enter data that disappears in the next update.’ (71).* *‘If we document client contact details in iCare’, it disappears, but if we update in e10, it will populate in iCare’ (80)* *‘Two different systems created by two different vendors, one is predominantly a finance system and the other is a clinical system. Those two vendors don’t talk to each other’ (79).*
		Lack of integration with external systems (e.g. My Health Record, government health system) for sharing information with hospital	*‘Link with government health system will help sharing information with doctors and hospitals’ (29).* *‘Integration to hospital systems so that there is automatic generation of task that can be checked off’ (3)*

### Theme 1: staff-related challenges

#### Staff behaviour and practices

Staff non-compliance and a poor attitude to documentation tasks were viewed as a main challenge, “*Some staff just don’t care [about documenting properly]” (1).* Second-hand documentation was also a common practice where staff would document client notes on behalf of others, which resulted in the omission of details, “*I request others to document on my behalf. So, I write only brief observation on a paper*” *(16)*. Verbal handover without updating information in clinical systems was also common. Although verbal handover is a standard health practice, it should be complimented by written documentation. One participant stated, “*I wish everything was [documented] in Clinical Manager for us, as things get forgotten at handover”* (*66*). Unintentional human error, such as forgetting to complete documentation and scanning, or entering data in incorrect forms and charts, was also acknowledged as a cause of data quality issues, “*We have a lot of documents to upload…the timeliness of info going into Clinical Manager….transfer of information from admission documents – human error can happen sometimes” (72)*.

#### Cultural diversity

A majority of participants reported that English language proficiency challenges among the aged care and disability services workforce contributed to poor quality of information documentation. “*There are so many free text fields where [the] quality of notes largely depends on the level of English of staff completing those fields” (84).*

#### Diversity in technical skills

There was consensus that the care workforce varied in terms of technical skills and that existing CIS do not “*appropriately engage the data-user at their level of knowledge” (32).* As explained by a direct care worker, *“A lot of us were not used to computers. We learned from bits and pieces and picking other people’s brain.” (64).*

#### Diversity in clinical knowledge

A majority of participants also reported that clinical knowledge varied across the workforce, including knowledge of clinical terminology and abbreviations, which impacted the quality of clinical documentation. A non-clinical participant stated, *“[A lot of] information is entered in the system which are summary text with acronyms and short-form [and] are difficult to understand. Information should be entered in a language that can be understood by other data-users*” *(81)*.

### Theme 2: education and training

#### Formal training in system use

A lack of structured and standardised training was reported to affect the quality of clinical data. Many participants reported learning to use the CIS informally, often through co-workers, “*Usually the person who is new…is shown how to use Clinical Manager through someone on site” (26)*, self-discovery, “*It wasn’t until I was thrown into this role that I just clicked on everything*” *(63)*, or previous training, “*[I was] trained in Clinical Manager elsewhere” (8).*

#### Role-specific training

Participants also reported a lack of role-specific education and training on how systems should be used by different data-user groups (e.g., nursing, allied health, care workers) and which groups are responsible for inputting specific information. “*People understanding their roles and responsibilities [impacts how up to date data and documentation are]” (25).*

### Theme 3: external barriers

#### Governance of external providers

External providers and agency staff are commonly employed to fill workforce gaps in aged care. Participants reported that external providers are not required to adhere to internal documentation policies which can impact data quality. “*External providers have their own book for notes… so we don’t know what happens” (41).*

#### Errors in health/medical documentation

Errors in external clinical documentation such as incorrect hospital discharge summaries were also common and are imported into CIS on admission. *“[There are often] incorrect information from hospital and [we] need to redo assessment” (5).*

#### Families as information source

Participants acknowledged the quality of initial data and documentation is often dependent on the quality of information provided by family members at admission. “*Sometimes family members don’t understand what we are trying to get out of them as admission information, which goes into better supporting clients, [so this information is missing]” (54).*

#### Sector-wide staff shortages

Participants frequently cited staff shortages as a cause of data quality issues. “*[Sites are] short staffed and therefore time is an issue” (1)*, and *“Staff are in a hurry, [there is a] shortage of staff [and] clients need attention” (40).*

### Theme 4: operational guidelines and procedures

#### Data and documentation requirements

Requirements for information documentation were unclear across the organisation and this impacted data quality. “*This is not just a systems problem;… [the organisation] needs to know [the data] requirements clearly to make [the] system provide what is needed instead of [collecting] all unnecessary details.” (29).*

#### Staff roles and responsibilities for data collection

Participants also reported a lack of clarity of roles and responsibilities for data collection across the organisation. *“Expectations for demographic data and accountability for roles [are] not clearly outlined or monitored” (32)*.

#### Staff roles and responsibilities for monitoring data collection

Lack of clarity around staff roles and responsibilities for monitoring data collection and quality was also reported. *“No one is monitoring [the] update of information [to ensure data is of high quality]” (63)*. A second participant added, “*who would monitor it anyway, who would start it, and who does training on it?” (34).*

### Theme 5: organizational practice and culture

#### ‘One size fits all’ approach

Participants reported that Clinical Manager is used across all residential sites and CareLink + for at-home services. However, the two systems are not able to meet the unique data and reporting requirements across all services. *“[Clinical Manager] may be working well for RAC but not for TCP” (1)* as operational requirements differ in these two service areas. Similarly, a participant from disability said, “*Clinical Manager is RAC focused, the care plans populate automatically in a way that is not appropriate for disability” (71)* as disability clients have functional impairments that are not captured through care plans focussed for aged care.

#### Workload and burden of documentation

Participants reported that the amount of documentation to be completed affected quality. *“If you have to write too many things, people copy and paste and it’s not always right” (40).* Relatedly, at larger sites, the volume of documentation required was often overwhelming, *“[There are] hundreds of progress notes… [It’s] too much” (59).* Direct care workload was also prioritised over documentation tasks, *“[Documentation] get completed at the end of the shift as we are busy, and often, [details] are forgotten at the end of the shift” (50).*

#### Communication of system or procedure changes to workforce

Participants also reported that changes to systems and documentation processes were not always effectively communicated to sites. *“[The organisation] doesn’t communicate changes when they make changes to processes.” (56).*

#### Duplication of effort

Duplication of effort in documentation was commonly reported and impacted data quality. Examples included doubling up of electronic and paper-based documentation, *“Some staff still use paper-based forms which takes time to be entered [electronically]” (82)*, storing documents in multiple locations *(37)*, and needing to enter the same information twice in different areas of the system, *“Forms don’t speak to each other and thus create duplication” (28).*

### Theme 6: Technological infrastructure challenges

#### System speed, connectivity, and reliability

System speed, connectivity and reliability at sites were important factors impacting the ability to document client notes in real-time. *“The system is slow. It plays a big part in me putting information into the system and there is a lag during busy times” (41).*

#### Technological support

Insufficient technical support was also identified as an important barrier to EHR use, *“[We] don’t know who to contact to fix bugs” (41).*

#### Equipment and resources

Participants expressed insufficient hardware at sites to support uninterrupted documentation at the point-of-care. *“Staff queue at the end of shift to document notes” (31).* Another participant stated, *“Technology has been the biggest issue, with constant glitches. One day iPads aren’t working [but] desktops are working, but there are not enough computers” (81).*

### Theme 7: systems design limitations

#### Data extraction and reporting

Participants reported major difficulties generating reports from the front-end systems, *“The function to pull out data [from Clinical Manager] is difficult, and it is difficult to work with generated reports” (49).* Similar difficulties were expressed by back-end data-users. *“The modelling of the data and tables at the back-end makes it difficult to pull data out for reporting. I think when these systems were created, they looked at it from function, and didn’t think about pulling data out for reporting” (79).*

#### Search and filter

Participants also expressed frustrations with the lack of inbuilt search functions to efficiently filter information. *“There is no search function to look for documents by keyword” (30).*

#### Single view of information

Participants reported frustrations with the lack of such single view of information, adding that they had to navigate through multiple screens to access information. *“Everything should be in one place rather than having to navigate through many places” (49).*

#### Change history

Participants expressed that timestamps and detailed change history are lacking which made it difficult to know whether client information was recent, *“There is no visible history of data being up-to-date” (31).* This made it difficult to keep track of who made changes to client information. *“I don’t think it’s possible to tell which field was updated by which person on this date” (81).*

### Theme 8: systems configuration

#### Dropdown values

Participants also reported frustrations with the configuration of dropdown menus, indicating that most values do not capture adequate clinical detail. *“[A client is] blind in the right eye for example – but the dropdown only has ‘sight/vision [issue]’. Dropdowns can limit and prevent person-centred care [by not allowing a sufficient level of clinical detail] (4)”.* Frustrations with choosing from long dropdown lists were also reported, *“It is difficult and time-consuming to understand what to choose from dropdown list (31)”.*

#### Free-text fields

Participants reported an overrepresentation of free-text fields which contributed to variation in data quality. *“There are so many free-text fields where the quality of notes largely depends on the level of English of staff” (84)* and *“Information is put in free-text fields, so it’s hard to be able to pull information out” (26).*

#### Automated information mapping

Participants expressed dissatisfaction with limited automation in CIS. *“Assessments don’t create alerts automatically and that’s why the reviews [are] forgotten when [staff] are busy” (2).* Where automation features were enabled, staff reported several errors in the configuration. *“[Adding a] second wound entry overwrites [the] active wound reminder” (60).*

#### Unused capabilities

Participants also believed that not all system capabilities were being used. *“There are more data in Clinical Manager that we could use but we don’t have training to be able to use all of its features” (51).*

#### Integration of multiple systems and information transfer

Finally, many participants reported frustrations with information transfer between systems. “*If we document client contact details in Clinical Manager, it disappears, but if we update in e10, it will populate in Clinical Manager” (80).* In addition to integration within internal systems, participants also commented on a lack of integration with external health data systems such as the Australian Government’s *My Health Record* system. “*Linking with the government health system will help share information with doctors and hospitals” (29).*

## Discussion

This study identified eight major causes of data quality issues in CIS at a large Australian aged care and disability services provider. Causes described were multi-faceted, combining both internal and external factors, and together highlight the complex environment surrounding the quality of clinical data and documentation in aged care and disability services. A coordinated and collaborative effort is required between service providers and the wider sector, to identify optimisation solutions which support safe and high-quality person-centred care and improved regulatory reporting.

The majority of causes identified in our study parallel barriers and facilitators previously identified in hospital settings, indicating that data quality challenges are prevalent across different healthcare environments. These include equipment and resource challenges, staffing and workload, role clarity for data collection, extraction, and monitoring, duplication of effort, standardisation of free-text documentation, communication of change, systems support, and staff training [7, 21, 27, 53, 54]. These challenges can be addressed through effective and clear clinical data governance policies and procedures and organisational leadership with a strong digital strategy. Adequate information technology infrastructure is the foundation of effective clinical documentation [54], which needs to be supported with formal staff education and training on the proper use of CIS in their day-to-day work [7, 25–27, 55]. Additionally, role clarity with clear workflows is essential to enhance performance and data quality [56].

Additional causes specific to aged care and disability services were identified which require solutions from a sector and/or policy level. Many of the identified causes related to systems design and configuration can be attributed to reliance on vendor-based “shrink-wrapped” or off-the-shelf systems modelled on designs from other healthcare environments. In particular, previous research has shown that it is not common practice for software vendors to engage clinician end-users in shaping the development of health information systems according to their preferences which contributes to system design limitations [57]. Moreover, as CIS systems are expensive to buy and maintain [58, 59], a common practice within the sector is to use a single CIS to meet multiple organizational requirements. Although cost-effective, a one-size fits all approach may not adequately support the workflows of multiple business areas [60]. Configuration of dropdown menus, automated alerts, information mapping, integration with other information systems, and building a single view of crucial information to meet the specific workflows and health information requirements of aged care and disability services is essential.

While systems can be configured to a degree at the organisation-level, a sector-level solution involving collaboration between service providers, vendors and regulatory bodies would promote standardised and systematic information capture and improved regulatory reporting across the sector. Standardisation would also facilitate data linkage [8] and information transfer with external CIS such as hospital and primary care and increase continuity of care. Effective integration of systems and information transfer is recognised as essential requirement of modern systems development [61].

National standardised CIS for aged care and disability services in Australia could also alleviate some of the burden associated with clinical documentation and regulatory reporting from individual service providers, especially amidst workforce shortages and increased information requirements with the expansion of the National Quality Indicator Program [62]. In 2022, a shortage of almost 100,000 aged care workers was estimated in Australia [63]. Workforce shortages underlie several causes of data quality issues described by participants in our study including reliance on external agency staff, and deprioritisation of documentation tasks, second-hand documentation, and verbal handover practices due to workload.

Effective CIS design for aged care and disability services in Australia also needs to be tailored to the cultural and technical diversity of the workforce. Almost a quarter (23%) of direct care workers in aged care have English as a second language [64]. The aged care workforce is also older than the overall workforce in Australia [64] and are likely to have lower levels of computer literacy [65]. Despite this, current CIS in aged care and disability services consist primarily of unstructured free-text fields, with the quality of documentation ultimately dependent on the English language and technical skills of the workforce. Unstructured free-text data is also difficult to analyse on a large-scale to derive meaningful insights [66].

Finally, prior research suggests that a combination of solutions that address both behavioural and technical factors are most effective in improving health information systems [20]. Well-designed CIS can support higher-quality clinical data by guiding the workforce to input the required information more efficiently but may not address all process-related barriers, which requires strong organisational leadership with clear and effective clinical data governance policies.

### Limitations

Study findings are limited to data-user experiences at a single service provider in WA and may not be generalisable to all aged care and disability services in Australia, particularly services which use different CIS than Clinical Manager and Carelink+. Moreover, although a balanced purposive sample of participants from all eight service areas were included, not all residential care facilities were consulted due to time and cost constraints. Likewise, convenience sampling was used which limited participation in this study to only a small sample of staff who were available at care sites at the time of visits. Finally, coder agreement measurement was not assessed.

## Conclusion

The quality of clinical data and documentation within aged care and disability services is influenced by a complex interplay of internal and external factors. While internal factors can be addressed to a degree by strong organisational leadership and governance, a coordinated and collaborative effort is ultimately required between service providers and the wider sector to identify optimisation solutions. Such solutions should aim to standardise the quality of clinical data to support safe and high-quality person-centred care and improved regulatory reporting.

### Electronic supplementary material

Below is the link to the electronic supplementary material.

Supplementary Material 1

## Data Availability

If interested in obtaining the datasets used or analysed in this study, please contact the corresponding author.
