# Supplementary material for: Factors impacting clinical data and documentation quality in Australian aged care and disability services: a user-centred perspective
Source: BMC Geriatr. 2024 Apr 12;24:338. doi: 10.1186/s12877-024-04899-1 (PMC11015693; doi:10.1186/s12877-024-04899-1)
Supplement: Supplementary file 1 — Supplementary Material 1 [file 12877_2024_4899_MOESM1_ESM.docx]

**Appendix 1**

1. What is your position or title?
   - *Administration*
   - *Clinical Psychologist*
   - *Community Support Worker*
   - *Disability Support Worker*
   - *Dietician*
   - *Enrolled Nurse*
   - *Occupational Therapist*
   - *Physiotherapist*
   - *Positive Behaviour Practitioner*
   - *Registered Nurse*
   - *Social Worker*
   - *Speech Pathologist*
   - *Support Coordinator*
   - *Therapy Assistant*
   - *Others ……………………………………………………………………………………..*
2. What service area do you work in?
   - *Brightwater At Home*
   - *Capacity Building*
3. How long have you worked for Brightwater?
   - *Less than 2 years*
   - *2-4 Years*
   - *More than 4 Years*
4. What is your experience of using iCare and/or Carelink?
5. We had a chance to go through 10% of client notes and found a number had information that was missing, incorrect, unclear, or not up to date. Why do you think this happens?
   - 1. Have you ever looked for certain client information in iCare/Carelink and found that it was missing or wasn’t recorded? Why?
     2. When looking at a client record in iCare/Carelink, how do you know whether the information is up to date? Do you know how regularly/often client information should be updated? What are some reasons why a client’s information might not be up to date?
     3. Have you ever found any incorrect information or had trouble understanding/interpreting any client information in iCare/Carelink? Why?
6. How easy is it to use client information from iCare/CareLink to support ……………………?
   - 1. *Clinical staff: Clinical decision making*
     2. *Care staff: Supporting client*
     3. *Backend users: Reporting and/or Analytics*
     4. *Service managers: Service management decisions and reporting*
     5. *Admin: Reporting*
7. What client information is crucial to record to provide person-centred care/reporting/analytics? Makes your job easier or not currently collected?
   - 1. *Site managers and clinical staff: reporting and delivering person-centred care?*
     2. *Care staff: delivering person-centred care?*
     3. *Corporate managers and backend users: reporting or analytics?*
8. Would you like to share anything else about iCare/CareLink?
